# A chronic strain of the cystic fibrosis pathogen *Pandoraea pulmonicola* expresses a heterogenous hypo-acylated lipid A

**DOI:** 10.1007/s10719-020-09954-8

**Published:** 2020-10-13

**Authors:** Molly D. Pither, Siobhán McClean, Alba Silipo, Antonio Molinaro, Flaviana Di Lorenzo

**Affiliations:** 1 Department of Chemical Sciences, University of Naples Federico II, via Cinthia 4, Naples, 80126 Italy; 2 Centre of Microbial Host Interactions, Institute of Technology Tallaght, Dublin, 24 Ireland; 3 School of Biomolecular and Biomedical Sciences, University College Dublin, Belfield, Dublin, 4 Ireland

**Keywords:** Lipopolysaccharide, Cystic Fibrosis, *Pandoraea*, Lipid A, Structural characterization, MALDI-TOF Mass spectrometry

## Abstract

*Pandoraea* sp. is an emerging Gram-negative pathogen in cystic fibrosis causing severe and persistent inflammation and damage of the lungs. The molecular mechanisms underlying the high pathogenicity of *Pandoraea* species are still largely unknown. As Gram-negatives, *Pandoraea* sp. express lipopolysaccharides (LPS) whose recognition by the host immune system triggers an inflammatory response aimed at the bacterial eradication from the infected tissues. The degree of the inflammatory response strongly relies on the fine structure of the LPS and, in particular, of its glycolipid moiety, i.e. the lipid A. Here we report the structure of the lipid A isolated from the LPS of a chronic strain of *P. pulmonicola* (RL 8228), one of the most virulent identified so far among the *Pandoraea* species. Our data demonstrated that the examined chronic strain produces a smooth-type LPS with a complex mixture of hypoacylated lipid A species displaying, among other uncommon characteristics, the 2-hydroxylation of some of the acyl chains and the substitution by an additional glucosamine on one or both the phosphate groups.

**Electronic supplementary material:**

The online version of this article (10.1007/s10719-020-09954-8) contains supplementary material, which is available to authorized users.

## Introduction

Cystic fibrosis (CF) is an incurable, chronic disease, caused by defects in genes encoding for the CF transmembrane conductance regulator (CFTR), a chloride channel which regulates the activity of other chloride and sodium channels at the cell surface epithelium [1]. Defective CFTR results in abnormal movement of salts and water across the cells which leads to dehydration of the airway surfaces and production of a thick and sticky mucus obstructing the pathways. This clog of the airways leads to breathing problems and to repeated and serious lung infections with damaging inflammation [2]. Indeed, the main cause of morbidity and mortality for CF patients is associated to infection by bacterial pathogens and opportunistic pathogens encountered throughout their lives. Beside *Pseudomonas aeruginosa* and bacteria of the *Burkholderia cepacia* complex, which are well-known Gram-negative opportunistic pathogens able to persistently colonize CF respiratory tract, *Pandoraea* species are considered as emerging pathogens leading to worsened CF lung disease [3].

Several species of *Pandoraea* have been identified so far, with *P. pulmonicola* representing the predominant species isolated in Irish CF patients. Moreover, *P. pulmonicola* is also considered as the most invasive and virulent among the *Pandoraea* species, exhibiting the capability to invade human lung epithelial cells, a peculiarity not shared by other *Pandoraea* species [4, 5]. However, still very little is known about the molecular mechanisms of pathogenicity and virulence of *Pandoraea* species as well as of their intrinsic multi-drug resistance [6].

A plethora of potential virulence factors have been previously defined for CF pathogens and connected with bacterial pathogenicity in CF. One of the most studied is the lipopolysaccharide (LPS) molecule which is the main component of the external leaflet of the Gram-negatives outer membrane. LPSs are crucial actors in a plethora of host-microbe interaction events such as, among others, colonization, adhesion, and virulence [7]. Importantly, the degree of pathogenicity of LPS molecules is strictly related to their ability to trigger the activation of the host innate and adaptive immune responses. Indeed, the LPS is recognized as a Pathogen-Associated Molecular Pattern (PAMP), i.e. a molecular signature relatively conserved among Gram-negatives, by specific Pattern Recognition Receptors (PRRs) of the host immune system. The receptorial complex made up of Toll-like receptor-4 (TLR4) and myeloid differentiation factor-2 (MD-2), present on the surface of several immune phagocytic cells, is one of the most studied PRRs which specifically recognizes the glycolipid moiety of an LPS [7]. Upon recognition by TLR4/MD-2, an intracellular signaling cascade is activated which culminates in the production of pro-inflammatory cytokines aimed at the clearance of the invading bacteria. However, this production of pro-inflammatory cytokines in immunocompromised individuals, as in the case of CF patients, is incredibly detrimental and leads to pathological consequences associated with high mortality [4, 5, 8].

The capability of an LPS to elicit such an inflammatory response is strictly related to its fine structure. In general, LPS is composed of three distinct domains: an outermost and highly variable polysaccharidic moiety (the O-chain) connected to an oligosaccharide (core OS) region which is, in turn, covalently linked to a glycolipid domain (the lipid A) which represents the anchor of the whole molecule to the outer membrane. The lipid A is the moiety specifically recognized by the TLR4/MD-2 complex and, depending on its structure, it can finely tune the degree of inflammatory cytokines release [9]. In general, a lipid A is made up of a β-(1→6) disaccharide of glucosamine (GlcN), typically phosphorylated at position 1 and 4’, and acylated by 3-hydroxy fatty acids in position 2, 2’, 3 and 3’ of the GlcNs. These acyl chains are, in turn, commonly acylated by other non-hydroxy fatty acids. Chemical modifications of such a general lipid A architecture greatly impact on the TLR4-mediated immunopotential of the whole LPS molecule. It is known that hexa-acylated lipid A with a 4 + 2 symmetry of the acyl chains with respect of the diglucosamine backbone, exhibits the strongest TLR4-mediated immune response, while tetra- and penta-acylated species have a weaker immunostimulatory capacity [7, 9]. Moreover, phosphate groups and additional decorations occurring on the glucosamine disaccharide backbone have a crucial role in the immunopotency of an LPS. In this context, several studies have been devoted to the characterization of the structure of lipid A from CF pathogens and opportunistic pathogens disclosing structural details involved in the bacterial pathogenicity and resistance to antibiotic treatment [10–13]. Moreover, as in the case of bacteria able to chronically colonize CF lungs, several lipid A structural modifications have been observed during the infection chronicization which can lower lipid A’s immunoelicitation power allowing enhanced persistence in the infected tissue [13].

Given these premises, the structural characterization of the lipid A moiety of an LPS remains a first but essential step in order to appreciate the molecular mechanisms underlying the LPS-mediated infection and inflammatory process observed in CF patients. Herein, we report about the structural elucidation of the lipid A from the LPS of *P. pulmonicola* strain RL8228, isolated from a chronically colonized CF Irish patient who died 52 months after their first *Pandoraea* isolation [5]. The structure has been defined by merging information that were attained from the compositional analysis executed on pure LPS and isolated lipid A with data from a matrix-assisted laser desorption ionization (MALDI) time of flight (TOF) mass spectrometry (MS) and MS^2^ investigation executed on the lipid A fraction and on the bacterial pellet.

## Methods

### LPS extraction and purification

The bacterial pellet was kindly provided by LMG collection of Ghent University. The LPS was extracted directly from dried pellet by the hot phenol-water procedure [14]. The LPS was then enzymatically digested, to remove possible cell contaminants, using DNase (DN25-Sigma Aldrich®, St. Louis, MO, USA), RNase (R5503-Sigma Aldrich®), and protease (P4630-Sigma Aldrich®). The digested material was extensively dialyzed (Spectra/Por®, cut-off 12–14 kDa) against distilled water and then ultracentrifuged (Beckman Coulter’s ultracentrifuge, Brea, CA, USA) (200,000 × *g*, 4 °C, 16 h). An additional step of purification foreseen a gel-filtration chromatography on a Sephacryl High Resolution S-400 (GE-Healthcare, Little Chalfont, UK) column. An SDS-PAGE followed by silver nitrate gel staining (Sigma Aldrich®, St. Louis, MO, USA) [15] was executed to ascertain the nature and the degree of purity of the extracted material.

### Compositional analyses of the fatty acid content

The total fatty acid content was determined by treating each LPS with 4 M HCl (100 °C, 4 h), followed by a treatment with 5 M NaOH (100 °C, 30 min). After the adjustment of the pH, fatty acids were extracted in CHCl_3_ and then methylated with diazomethane and analyzed by Gas Chromatography Mass Spectrometry (GC-MS) (Santa Clara, CA, USA). The ester-bound fatty acids, analyzed by GC-MS, were recovered after treatment with aqueous 0.5 M NaOH in CH_3_OH (1:1, *v/v*, 85 °C, 2 h), followed by acidification of the products, extraction in CHCl_3_ and methylation with diazomethane.

In parallel, an aliquot of each LPS fraction was also methanolized with 1.25 M HCl/CH_3_OH (80 °C, 16 h). The mixture was extracted three times with hexane. The hexane layer, containing the fatty acids as methyl esters derivatives, was then analyzed by GC-MS.

The absolute configuration of the fatty acids was defined as previously reported in literature [16]. Briefly, the 3-hydroxy fatty acids were released after treatment with 4 M NaOH (100 °C, 5 h), converted into the 3-methoxy acid L-phenylethylamides, and then analyzed by GC-MS. The comparison of the retention times of authentic L-phenylethylamides of various standard fatty acids with those from the *P. pulmonicola* LPS led the assignment of the (*R*) configuration to 14:0 (3-OH) acyl moieties and the (*S*) configuration to 14:0 (2-OH) and 12:0 (2-OH). The analyses were all performed on an Agilent Technologies gas chromatograph 6850A equipped with a mass selective detector 5973N and a Zebron ZB-5 capillary column (Phenomenex, 30 m × 0.25 mm internal diameter, flow rate 1 mL min^−^1, He as carrier gas). The following temperature program was employed for the lipid analysis: 140 °C for 3 min, 140 °C → 280 °C at 10 °C min^− 1^.

### Isolation, chemical analysis, and partial de-lipidation of the lipid A fraction

An aliquot of purified LPS was treated with acetate buffer (pH 4.4, 2 h, 100 °C) in presence of SDS, in order to selectively cleave the lipid A from the saccharide part of the LPS. A mixture of CHCl_3_ and CH_3_OH was added to the hydrolysis product to obtain a CHCl_3_/CH_3_OH/hydrolysate 2:2:1.8 (*v/v/v*) ratio. The mixture was then shaken and centrifuged. The chloroform phase, containing the lipid A, was collected and washed with the water phase of a freshly prepared Bligh/Dyer mixture (CHCl_3_/CH_3_OH/water, 2:2:1.8) [17]. The organic phases, containing the lipid A fraction, were pooled and dried. The sample preparation methods reported here fulfill the Minimum Information Required for a Glycomics Experiment (MIRAGE) guidelines [18].

In order to establish the nature of the sugar(s) composing the backbone of the lipid A, an aliquot of the lipid A fraction also underwent a methanolysis (1.25 M HCl/CH_3_OH, 80 °C, 16 h) followed by acetylation (80 °C, 20 min) and GC-MS analysis [19, 20].

To partially remove the acyl chains, an aliquot of lipid A fraction (0.3 mg) was treated with ammonium hydroxide (NH_4_OH) as previously reported [21]. The sample was then dried and analyzed by MALDI-TOF MS along with the untreated lipid A fraction.

### Dephosphorylation of lipid A and derivatization to alditol acetates

In order to unequivocally establish the nature of the additional hexosamine(s) decorating the lipid A, around 0.6 mg of the mild acid hydrolysis product, i.e. the isolated lipid A fraction, was treated with 100 µL of 48% aqueous hydrofluoric acid (HF) (Sigma Aldrich®, St. Louis, MO, USA) at 4° C for 16 h to remove the phosphate groups. The sample was placed in an ice bath and the HF was then evaporated util dry. The HF-treated sample was then dissolved in distilled water and lyophilized. A mixture of CHCl_3_/CH_3_OH/H_2_O (2:1:2 *v/v/v*) was added to the sample and the upper part was collected and lyophilized [22]. The sample was then treated with NaBH_4_ (Sigma Aldrich®, St. Louis, MO, USA) and then acetylated with equal amounts of acetic anhydride in pyridine at 85 °C for 20 min [20]. The so-obtained acetylated alditol derivative of the isolated hexosamine was analyzed by GC-MS and compared with opportunely prepared standards.

### MALDI-TOF mass spectrometry

All the MS and the MS^2^ experiments were performed both in linear and reflectron mode, negative ion polarity on an ABSCIEX TOF/TOF 5800 Applied Biosystems (Foster City, CA, USA) mass spectrometer equipped with an Nd:YAG laser (λ = 349 nm), with a 3 ns pulse width and a repetition rate of up to 1000 Hz, and also equipped with delayed extraction technology. Lipid A fraction was dissolved in CHCl_3_/CH_3_OH (50:50, *v/v*). The matrix solution was 2’,4’,6’-trihydroxyacetophenone (THAP) (91,928-Sigma Aldrich®) in CH_3_OH/0.1% trifluoroacetic acid/CH_3_CN (7:2:1, *v/v/v*) at a concentration of 75 mg/mL [23–25]. The NH_4_OH-treated lipid A was instead dissolved in CHCl_3_-trifluoroethanol (4:1, *v/v*) and the matrix used was 2,5-dihydroxy benzoic acid (DHB) (85,707-Sigma Aldrich®) in acetonitrile 0.2% trifluoroacetic acid (7:3, *v/v*) [21]. In the case of analysis of the bacterial pellet, the matrix used was DHB at a final concentration of 10 mg/mL in CHCl_3_/CH_3_OH (9:1, *v/v*) as previously reported [26]. In all the cases, 0.5 µL of the sample and 0.5 µL of the matrix solution were deposited onto a stainless-steel plate and left to dry at room temperature. Each spectrum in the MS experiments was a result of the accumulation of 2000 laser shots (raw file was uploaded to Glycopost (GPST000137 https://glycopost.glycosmos.org/), whereas 5000–7000 shots were summed for the MS^2^ spectra. Each experiment was performed in triplicate.

## Results

### Isolation of the LPS and compositional analysis of the lipid A from *P. pulmonicola* strain RL8228

The LPS material was isolated from lyophilized bacterial cells and checked via SDS-PAGE after silver nitrate gel staining [14, 15]. This analysis showed that *P*. *pulmonicola* RL8228 expresses an S-LPS as proven by the ladder-like pattern in the upper part of the gel, indicative of the presence of the O-chain moiety. After purification of the LPS, a detailed compositional analysis was performed to establish the fatty acid content, revealing the occurrence of (*R*)-3-hydroxytetradecanoic acid (14:0 (3-OH)) in both ester and amide linkages, whereas (*S*)-2-hydroxytetradecanoic (14:0 (2-OH)), (*S*)-2-hydroxydodecanoic (12:0 (2-OH)), dodecanoic (12:0) and tetradecanoic acid (14:0) were found only as ester-bound acyl chains.

In order to elucidate the structure of the lipid A moiety, an aliquot of the LPS underwent a mild acid hydrolysis, which selectively cleaves the acid labile glycosidic linkage bridging the core OS with the lipid A portion. Once obtained the lipid A fraction, an aliquot underwent a compositional analysis to define the nature of the lipid A sugar backbone. This analysis revealed the occurrence of GlcN as the only sugar composing the lipid A moiety.

In parallel, another aliquot of the LPS mild acid hydrolysis product underwent a detailed MALDI-TOF MS and MS^2^ analysis to establish the fine structure of the lipid A. Finally, in order to avoid any loss of structural information, a MALDI-TOF investigation of the lipid A was also executed directly on the bacterial pellet, as previously described [26].

### MALDI-TOF MS and MS^2^ analysis on the isolated lipid A from *P. pulmonicola* strain RL8228

**Fig. 1:**
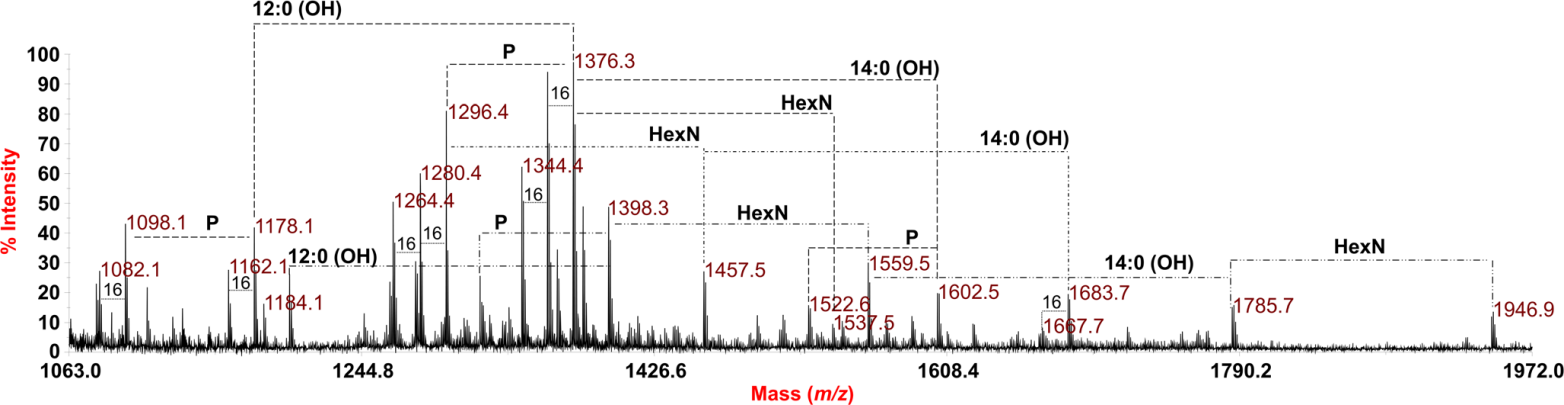
Negative-ion MALDI-TOF (reflectron mode) mass spectrum of the lipid A from *P. pulmonicola* RL8228 obtained by mild acid treatment of the purified LPS. Differences of 16 amu are reported in the spectrum. Differences in the nature of the acyl chains have been also indicated. “**P**” indicates the phosphate group; “**HexN**” indicates differences of 161 amu (i.e. a hexosamine unit)

The reflectron MALDI-TOF MS spectrum, recorded in negative ion polarity, of the isolated lipid A from *P. pulmonicola* RL8228 is reported in Fig. 1. The spectrum clearly showed an extremely complex pattern of signals relative to deprotonated [M-H]^−^ lipid A species differing in the nature and number of the fatty acid chains and in the phosphate content. Moreover, the spectrum displayed additional peaks differing in 161 amu, which likely suggested the occurrence of lipid A species characterized by hexosamine (HexN) modification on one or both phosphate groups of the disaccharide backbone (Fig. 1; Table 1).

**Table 1:** The main ion peaks observed in the MALDI-TOF MS spectrum reported in Fig. 1, the predicted mass and the proposed interpretation of the substituting fatty acids and phosphates on the *P. pulmonicola* RL8228 lipid A backbone

Predicted mass (Da)	Observed ion peaks (*m*/*z*)	Acyl substitution	Proposed fatty acid/phosphate composition
1098.69	1098.16	Tri-acyl	HexN^2^P [14:0(3-OH)]^3^
1178.66	1178.16	Tri-acyl	HexN^2^P^2^ [14:0(3-OH)]^3^
1296.86	1296.41	Tetra-acyl	HexN^2^P [14:0(3-OH)]^3^[12:0(2-OH)]
1376.82	1376.35	Tetra-acyl	HexN^2^P^2^ [14:0(3-OH)]^3^[12:0(2-OH)]
1456.92	1457.48	Tetra-acyl	HexN^3^P [14:0(3-OH)]^3^[12:0(2-OH)]
1523.05	1522.62	Penta-acyl	HexN^2^P [14:0(3-OH)]^3^[12:0(2-OH)] [14:0(2-OH)]
1537.89	1537.50	Tetra-acyl	HexN^3^P^2^ [14:0(3-OH)]^3^[12:0(2-OH)]
1603.02	1602.59	Penta-acyl	HexN^2^P^2^ [14:0(3-OH)]^4^[12:0(2-OH)]
1684.12	1683.75	Penta-acyl	HexN^3^P [14:0(3-OH)]^4^[12:0(2-OH)]

The observed masses reported in the table are compared to the calculated molecular weight (predicted mass, Da) of each ion based on the proposed lipid A structures.

Briefly, *mono*- and *bis*-phosphorylated tri- to penta-acylated lipid A species have been identified in the mass range *m/z* 1082.1–1946.9, with a visibly clear high heterogeneity due also to differences of 16 amu occurring between most of the peaks, indicative of lipid A species differing in the absence or presence of hydroxylated acyl chains. The main peak at *m/z* 1376.3 was matched with a *bis*-phosphorylated tetra-acylated lipid A species carrying two primary *N*-linked 14:0 (3-OH), one *O*-linked primary 14:0 (OH), and one secondary 12:0 (2-OH), whose *mono*-phosphorylated form was detected at *m/z* 1296.4 (Fig. 1; Table 1). Moreover, the related *bis*-phosphorylated penta-acylated lipid A form bearing an additional hydroxylated 14:0 moiety with respect to species at *m/z* 1376.3, matched with peak at *m/z* 1602.5 (Table 1). Interestingly, starting from the *mono*-phosphorylated tetra-acylated lipid A species at *m/z* 1296.4, it was possible to identify a related species decorated by one additional HexN (differing for 161 amu) matching with peak at *m/z* 1457.5; the corresponding penta-acylated form, carrying another secondary 14:0 (2-OH), was identified at *m/z* 1683.7 (Fig. 1; Table 1).

Furthermore, the spectrum showed an additional series of peaks which are not directly related to any other known modifications of the lipid A but predicted to belong to lipid A as the differences between them (198 and 80 amu from *m*/*z* 1398.3 to *m*/*z* 1200.1, and to *m/z* 1318.4) can be attributed to one 12:0 (OH) and one phosphate group, respectively. Moreover, starting from the lipid A species at *m/z* 1398.4, additional lipid A forms were identified as decorated by one HexN (*m/z* 1559.5), one HexN and one 14:0 (OH) (*m/z* 1785.7), and two HexNs and one 14:0 (OH) (*m/z* 1946.9). Notably, the negative-ion MALDI-TOF mass spectrum, which was recorded directly on the intact bacterial cells (Fig. S-1), confirmed the structural determination deduced by analysis of the isolated lipid A fraction (Fig. 1). This observation consolidated the results and excluded any lack of structural information possibly occurring as a consequence of the chemical treatment to isolate the lipid A.

**Fig. 2:**
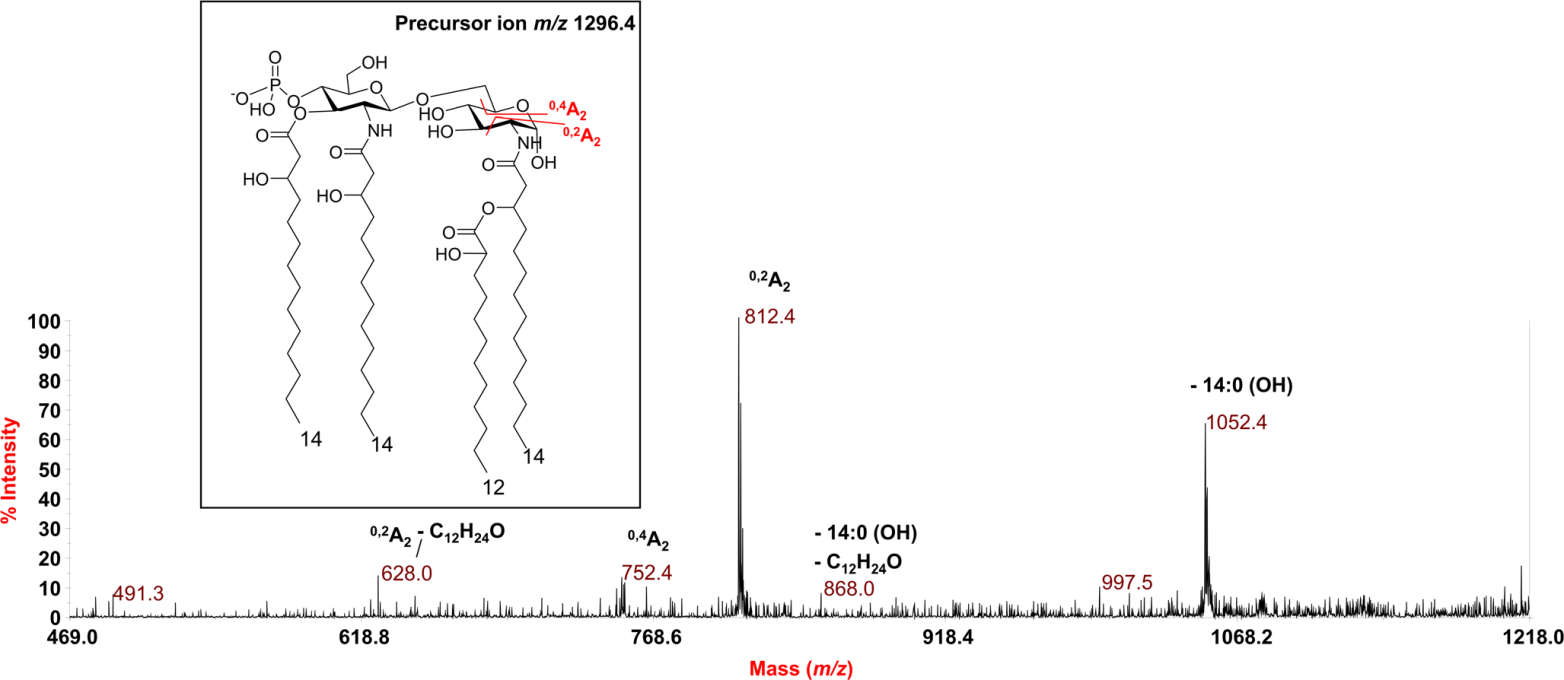
Negative-ion MALDI MS^2^ spectra of precursor ion at *m/z* 1296.4 of the lipid A isolated from. *P. pulmonicola* RL8228. This is a representative ion peak of the cluster ascribed to tetra-acylated lipid A species decorated by one phosphate. The main fragments’ assignment is indicated in the spectrum. The proposed structure is reported in the inset with the observed sugar ring fragmentations (^0,4^A_2_ and ^0,2^A_2_). The loss of C_12_H_24_O (184 mass units) is also indicated and was due to a rearrangement typically occurring on primary 14:0 (3-OH) acyl chains only when their 3-OH group is free, thus contributing to the establishment of the location of the secondary acyl substitution

In order to meticulously delineate the structure of *P. pulmonicola* RL8228 lipid A, that is defining the exact location of the acyl chains, phosphates and additional HexN with respect to the diglucosamine backbone, a negative-ion MS^2^ analysis was performed on various peaks. The MS^2^ spectrum of precursor ion at *m/z* 1296.4 (Fig. 2), chosen as a representative of *mono*-phosphorylated tetra-acylated lipid A species, showed an intense peak at *m/z* 1052.4 attributed to an ion derived from the loss of one primary 14:0 (3-OH). However, an important peak, in terms of structural characterization, was identified at *m/z* 812.4 which was attributed to an ion originating from the sugar ring fragmentation ^0,2^A_2_ [27] which demonstrated that **(i)** two hydroxylated 14:0 chains and the phosphate decorated the non-reducing glucosamine unit, and suggested **(ii)** that the hydroxyl group at position 3 of the reducing glucosamine was free; this, in turn, proved that **(iii)** the 12:0 (2-OH) acyl chain was present as a secondary substituent of the primary amide-bound 14:0 (3-OH) of the reducing glucosamine. A further sugar ring fragmentation (^0,4^A_2_) [27] at *m/z* 752.4 was also identified, and concurred to confirm the above structural hypothesis.

**Fig. 3:**
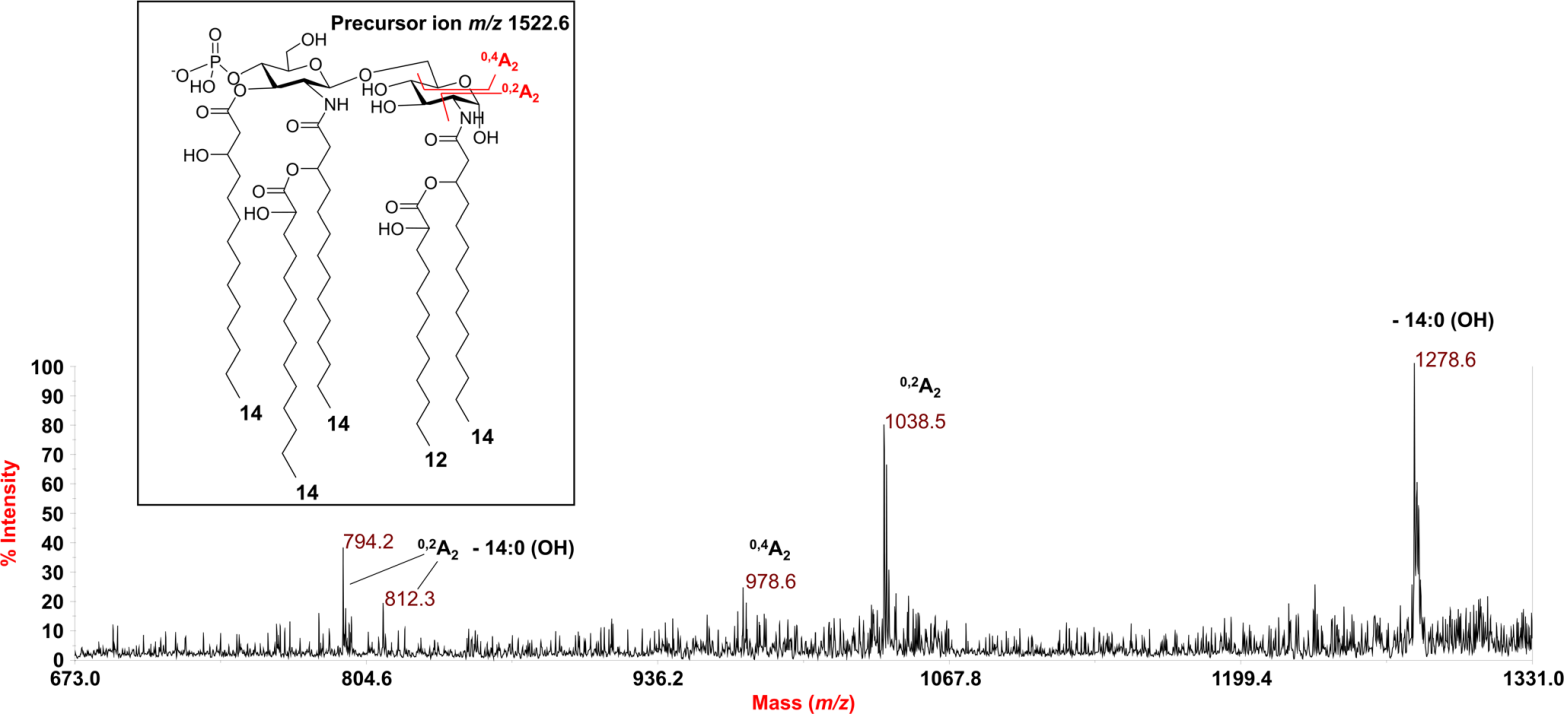
Negative-ion MALDI MS^2^ spectrum of precursor ion at *m/z* 1522.6 of the lipid A isolated from. *P. pulmonicola* RL8228. This is a representative ion peak of the cluster attributed to penta-acylated lipid A species decorated by one phosphate. The main fragments’ assignment is indicated in the spectrum. The proposed structure is given in the inset with the observed sugar ring fragmentations (^0,4^A_2_ and ^0,2^A_2_)

The negative-ion MS^2^ spectrum of precursor ion at *m/z* 1522.6 (Fig. 3), identified as a *mono*-phosphorylated penta-acylated lipid A species, was chosen to settle the location of the secondary acyl moieties. In detail, the MS^2^ spectrum showed an intense peak at *m/z* 1278.6 matching with an ion derived from the loss of one hydroxylated 14:0 moiety (Fig. 3). In addition, the peak at *m/z* 1038.5, assignable to an ion originating from the sugar ring fragmentation ^0,2^A_2_, proved that the non-reducing glucosamine unit was decorated by three acyl chains, that is plausibly two primary 14:0 (3-OH) and one secondary 14:0 (2-OH). The absence of fragments matching with the loss, from the precursor ion, of a whole unit of a hydroxylated 14:0 fatty acid carrying a secondary hydroxylated 14:0 unit, suggested that the secondary acyl substitution occurred on the *N*-linked primary acyl chain. Likewise, the absence of a peak matching with an ion derived from the sequential loss of one 14 (OH) and one 12 (OH), suggested that the latter was in an acyloxyacyl moiety. Finally, and further supporting the structural assessment, the peaks at *m/z* 812.3 and *m/z* 794.2 were assigned to the sugar ring fragmentation ^0,2^A_2_ plus the loss of one hydroxylated 14:0, likely the primary ester-bound acyl moiety, eliminated as either a free fatty acid (*m/z* 794.2) and as a ketene derivative (*m/z* 812.3).

**Fig. 4:**
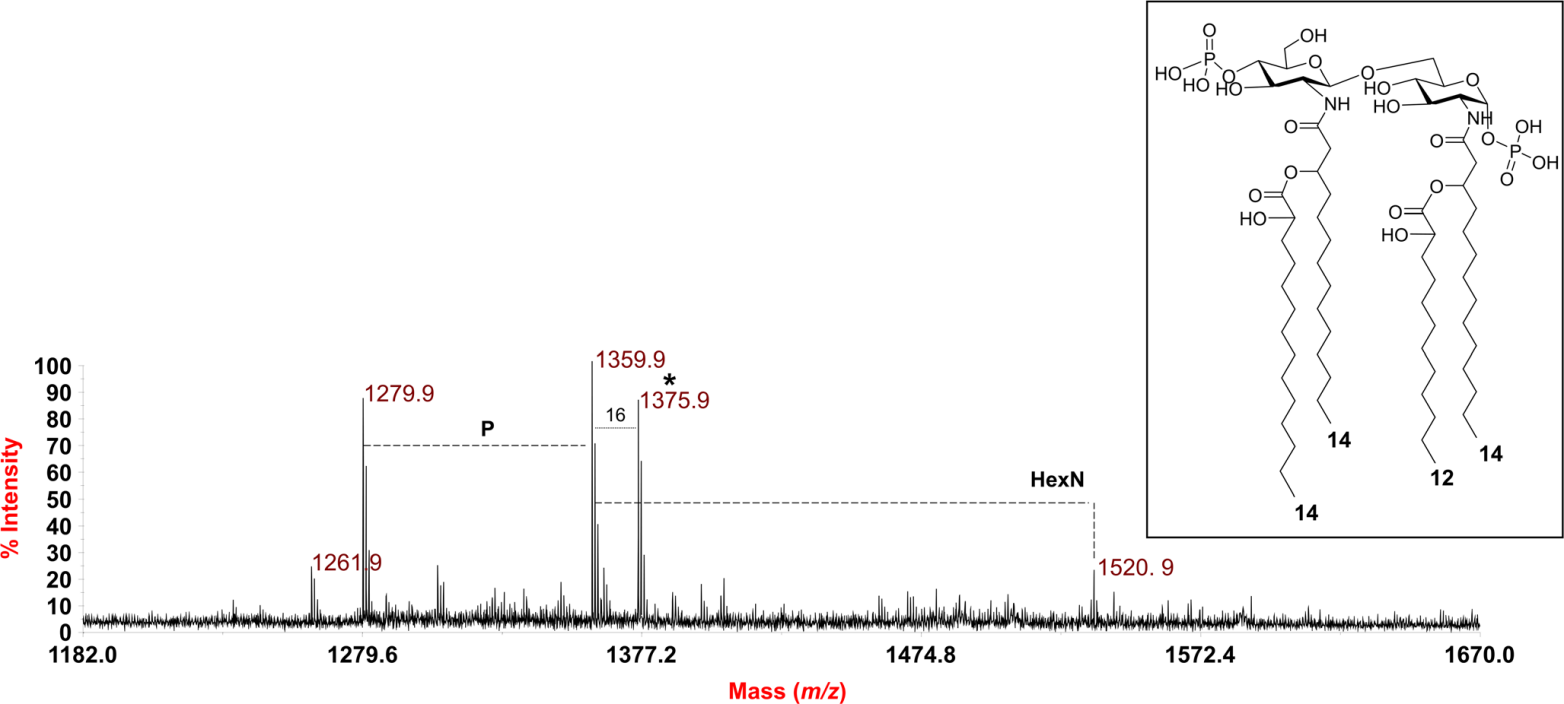
Negative ion MALDI-TOF (reflectron mode) mass spectrum of the lipid A after NH_4_OH hydrolysis. *Indicates the structure sketched in the inset. “**P**” indicates the phosphate group; “**HexN**” indicates differences of 161 amu (i.e. a hexosamine unit)

Since the data from MS^2^ analyses led to locate the secondary acyl substitution only on the primary amide-bound fatty acids, an aliquot of lipid A was subjected to a treatment with NH_4_OH [21]; this procedure by selectively removing the acyl and acyloxyacyl esters, while leaving the acyl and acyloxyacyl amides unaltered, is typically crucial in providing a clear indication of the location of the lipid A acyl chains. The MALDI-TOF MS spectrum, recorded in negative polarity, of the NH_4_OH-treated lipid A is reported in Fig. 4. It clearly showed three main peaks at *m/z* 1279.9, 1359.9 and 1375.9 matching with *mono*- (*m/z* 1279.9) and *bis*-phosphorylated (*m/z* 1359.9 and 1375.9) tetra-acylated lipid A species carrying the sole primary *N*-linked hydroxylated 14:0 acyl chains in turn substituted by the secondary fatty acids, which were unaffected by the NH_4_OH treatment. This definitively proved that the secondary acyl substitution in the lipid A from *P. pulmonicola* RL8228 exclusively occurs on the primary amide-bound fatty acids.

**Fig. 5:**
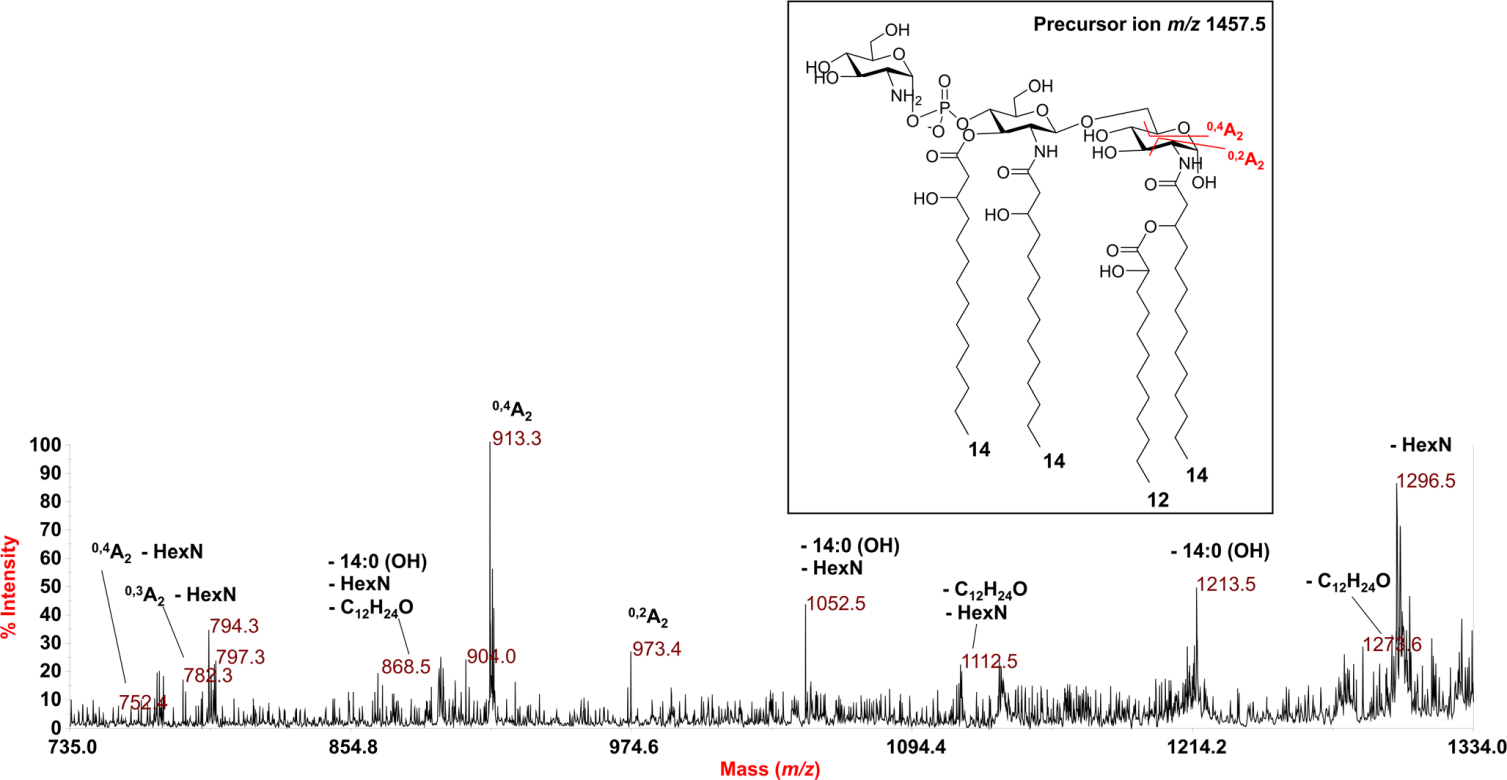
Negative-ion MALDI MS^2^ spectrum of precursor ion at *m/z* 1457.5 of the lipid A isolated from *P. pulmonicola* RL8228. This is a representative ion peak of the cluster ascribed to tetra-acylated lipid A species decorated by one phosphate and one hexosamine via phosphodiester bridge. The main fragments’ assignment is indicated in the spectrum. The proposed structure is reported in the inset with some of the observed sugar ring fragmentations (^0,4^A_2_ and ^0,2^A_2_). The α configuration at the anomeric center of the GlcN unit was tentative. “**HexN**” indicates differences of 161 amu (i.e. a hexosamine unit)

In conclusion, to shed light on the actual presence of lipid A species decorated by additional HexN(s) linked to the diglucosamine backbone via the phosphate group(s), the precursor ion at *m/z* 1457.5 was analyzed by negative-ion MS^2^. The spectrum (Fig. 5) showed an intense peak at *m/z* 1296.5 attributed to an ion derived from the loss of a HexN unit, whereas peak at *m/z* 1213.5 was assigned to an ion generated by the loss of one hydroxylated 14:0 moiety. The occurrence of the peak at *m/z* 913.3, identified as an ion derived from the sugar ring fragmentation ^0,4^A_2_, proved that the additional HexN unit was on the phosphate decorating the non-reducing glucosamine unit, in turn acylated by two hydroxylated 14:0 residues. In parallel, peak at *m/z* 973.4 (^0,2^A_2_) confirmed the location of the additional HexN as well as the location of the secondary acyl chain (12:0 (2-OH)) in the acyloxyacyl amide moiety of the reducing glucosamine unit (Fig. 5). Moreover, at low molecular masses an ion matching with HexN-PO_3_ (*m/z* 240.12) has been also detected (Fig. S-2). In conclusion, despite chemical analyses only showed the presence of GlcN in the isolated lipid A fraction, in order to unequivocally define the nature of the additional HexN decorating some lipid A species of *P. pulmonicola* RL8228 LPS, an aliquot of lipid A underwent a dephosphorylation treatment; this was followed by derivatization to alditol acetates of the unknown HexN properly separated from the lipid A moiety, which was then investigated by means of GC-MS (Fig. S-3). This analysis revealed the occurrence of glucosaminitol acetate, thus finally demonstrating that the additional HexN unit decorating the disaccharide backbone of some lipid A species from *P. pulmonicola* RL8228 was a GlcN.

## Discussion

Chronic lung infection by *Pandoraea* species is an emerging concern for the care and treatment of CF patients. Indeed, after a first colonization, *Pandoraea* species are not only able to chronically colonize the respiratory tract of CF individuals, but they are also easily transmitted between patients causing an aggravation of the lung disease and bacteremia [28]. In this frame, *P. pulmonicola* strains have been identified as the most virulent among the other *Pandoraea* species reported so far, both in *in vitro* and *in vivo* models [5]. This has been associated to their capability to invade lung tissues and to their resistance to a broad spectrum of antibiotics commonly used for the treatment of bacterial infections in CF. However, the contribution of *P. pulmonicola* to pathology progression in CF patients is not fully understood. In large measure, this lack of information is due to the still scant reports in clinical and research literature outlining their colonization of CF lung and their associated virulence factors.

**Fig. 6:**
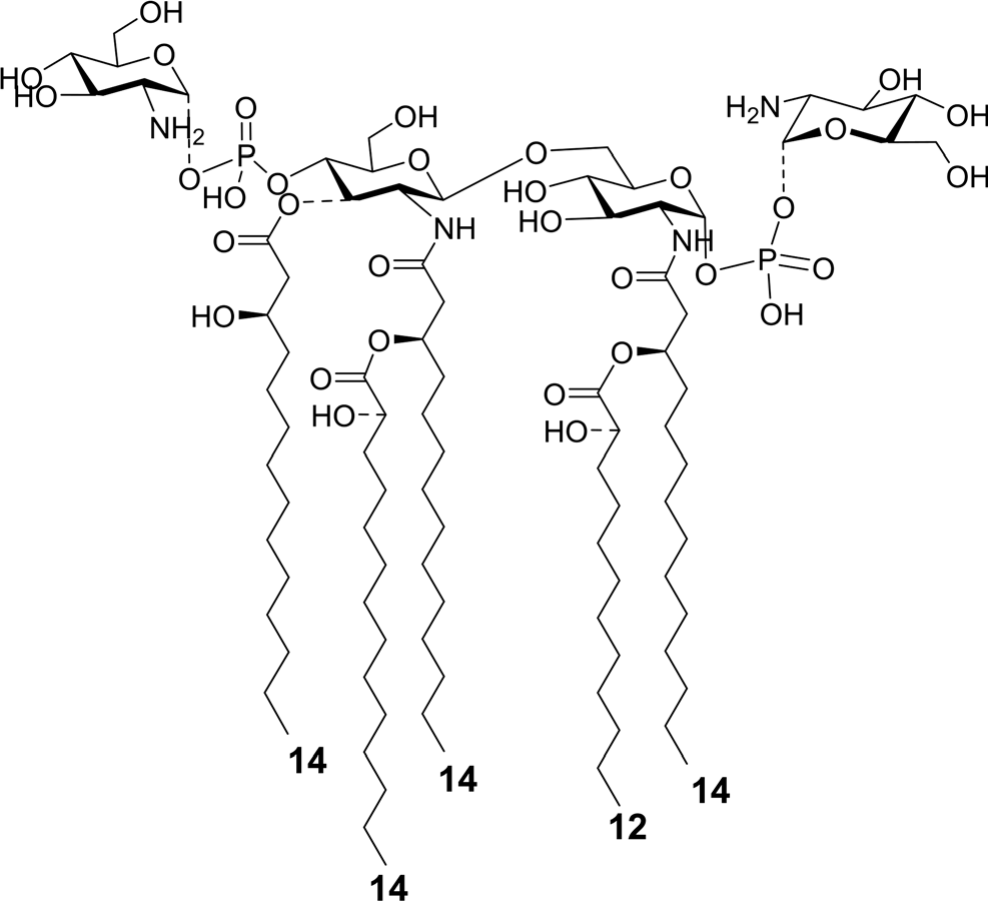
Summary of the variable structural features observed for *P. pulmonicola* RL8228 lipid A. Some of the observed incompleteness of the substitutions are reported as dotted-line bonds. The secondary acyl moieties can also be non-hydroxylated and the possible occurring fatty acids have been indicated as suggested also by chemical analyses data. The α configuration at the anomeric center of the GlcN units was tentative

To gain an insight into the potential virulence mechanisms of *P. pulmonicola*, we focused the attention on the main constituent of its outer membrane, the LPS molecule. As a potent elicitor of the host immune system, the LPS, and in particular its lipid A moiety, surely has a role in the virulence of this bacterium. Here we characterized the lipid A structure isolated from the smooth-type LPS of the chronic strain *P. pulmonicola* RL8228. The lipid A turned out to be a complex mixture of species differing by nature and number of the acyl chains, but also for the phosphate content and the additional decoration on one or both the phosphate groups with GlcN. Briefly, tri- to penta-acylated lipid A species carrying one or two phosphates have been identified, decorated by hydroxylated 14:0 as primary acyl chains, while various secondary fatty acids (i.e. 12:0 (2-OH), 14:0 (2-OH), 14:0 and 12:0) were found (Fig. 6). In particular, the main lipid A species, detected at *m/z* 1376.4, was matched with a *bis*-phosphorylated tetra-acylated form carrying two primary hydroxylated 14:0 units on the non-reducing glucosamine, and one primary *N*-linked hydroxylated 14:0 in turn substituted by the secondary 12:0 (2-OH) fatty acid on the reducing glucosamine. Lipid A species decorated by an additional hydroxylated 14:0, yielding the penta-acylated forms were also identified. Furthermore, as stated above, lipid A species decorated by one or two GlcN residues linked via phosphodiester bonds have been detected. A visual summary of all the structural variations observed in such a complex lipid A blend is reported in Fig. 6.

Interestingly, the structural elucidation of the sole lipid A from *P. pulmonicola* RL8228 S-LPS already revealed several structural features that could give, although preliminary, an explanation to the exceptional virulence of this strain as well as its capability to chronicize in CF lungs. Indeed, **(i)** the capability to express different lipid A species simultaneously with varying abundance, **(ii)** the low degree of acylation (less than six acyl chains), **(iii)** the unusual fatty acid asymmetry and variability at position 3 and 3’, **(iv)** the 2-hydroxylation of both 14:0 and 12:0 in the acyloxyacyl positions at 2 and 2’, as well as **(v)** the occurrence of the additional decoration by GlcN of the phosphate groups, are surely of note for this lipid A structure. Most of these characteristics are considered as a trick adopted by bacteria to escape the host immune surveillance and thus enabling their persistence in the infected tissue. Indeed, it is known that some lipid As with less than six acyl chains are able to only poorly induce the TLR4 activation and resulting responses, thus avoiding the anti-bacterial effects derived from the induction of pro-inflammatory cytokines production [9]. Moreover, the presence of positively charged substituents, that is the GlcN, on the phosphate groups, “neutralizing” their negative charge, is known to strengthen bacterial resistance to host-derived cationic antibacterial peptides, but also to reduce bacterial susceptibility to penetration by commonly used cationic antibiotics [10–13]. Also, it has been demonstrated, for other CF pathogens, that the 2-hydroxyacylation of the lipid A is involved in abrogation of the innate immunity inflammatory response, thus rendering the bacterium less prone to be cleared from the lungs [29]. This is plausibly due to the fact that the presence of additional hydroxylated acyl chains, in place of non-hydroxylated ones as secondary substituents, may result in an increment of the H bonding between neighboring LPS molecules in the bacterial outer membrane, thereby incrementing the stabilization of the membrane itself and likely increasing resistance to antibiotic/antimicrobial peptides-induced bacterial killing.

Notably, some structural features found for the *P. pulmonicola* RL8228 lipid A resemble those observed for another respiratory tract pathogens, i.e. *Bordetella* sp. Indeed, a similar fatty acid composition and distribution with the respect to the glucosamine disaccharide backbone has been observed for some *Bordetella* species, in addition to the occurrence of the additional glucosamine substituting one or both lipid A phosphate groups [30]. However, in contrast to *P. pulmonicola* RL8228, in *Bordetella* sp. short-chain fatty acids (10:0 (OH)) are commonly found, in addition to the occurrence, in some strains, of 16:0 as a modification required for persistent colonization of the mouse respiratory tract, [31] and for protection from complement-mediated killing during *Bordetella* respiratory infection [32].

How and at what extent the above structural features determined for *P. pulmonicola* RL8228 lipid A are involved in the pathogenicity of such an emerging CF pathogen is currently under investigation in the context of a detailed structure to function relationship study.

## Conclusions

This is the first report on the structure of the LPS lipid A moiety isolated from *Pandoraea* species. Here we investigated the structure of the lipid A from the chronic strain *P. pulmonicola* RL8228. This highly virulent CF pathogen showed to express a complex blend of lipid A species which differ in both the phosphate content, the nature and number of the acyl chains. Furthermore, some lipid A species have been found decorated by additional GlcN via phosphodiester bridge. The combination of the structural features observed in the lipid A are likely connected to the capability of this strain to persist in the CF lungs avoiding detection by the host immune system as well as its disruption by the most commonly used antibiotics. Similarly, we have previously characterized the O-chain structure of the LPS isolated from another *P. pulmonicola* strain (LMG 18,108) and demonstrated that it was made up of the trisaccharide repeating unit [→2-β-D-Qui3N-(1→4)-α-D-GalNAc-(1→3)-α-D-GlcNAc-1→] where a five-membered ring aglycon residue has been found covalently linked to the amine group of Qui3N [33]. As the O-chain is the antigenic portion of the LPS, conferring the uniqueness indispensable for antibody recognition, the occurrence of this non-sugar appendage was hypothesized to have a role in preventing the recognition of the bacterium by host immune system. In this perspective, future studies are currently ongoing to define the full structure of the saccharide component of the LPS from *P. pulmonicola* RL8228 and the immunological properties of the whole molecule.

## Electronic supplementary material

ESM 1 (PDF 433 kb)

## Data Availability

The datasets used and/or analyzed during the current study are available from the corresponding author on reasonable request.

## Abbreviations

CF: Cystic Fibrosis
CFTR: Cystic Fibrosis transmembrane conductance regulator
DHB: Dihydroxy benzoic acid
GC-MS: Gas Chromatography-Mass Spectrometry
HexN: Hexosamine
HF: Hydrofluoric acid
LPS: Lipopolysaccharide
MALDI: Matrix-Assisted Laser Desorption Ionization
PAMP: Pathogen Associated Molecular Pattern
MD-2: Myeloid Differentiation factor-2
MS: Mass Spectrometry
OS: Oligosaccharide
PRR: Pattern Recognition Receptor
SDS-PAGE: Sodium Dodecyl Sulfate-Polyacrylamide Gel Electrophoresis
S-LPS: Smooth-type Lipopolysaccharide
THAP: 2′,4′,6′-Trihydroxyacetophenone
TLR4: Toll-Like Receptor 4
TOF: Time Of Flight

## References

[1] Rafeeq MM, Murad HAS (2017). Cystic fibrosis: current therapeutic targets and future approaches. J. Transl. Med..

[2] Sockrider MM, Ferkol TW (2017). Twenty Facts About Cystic Fibrosis. Am. J. Respir. Crit. Care Med..

[3] Mahenthiralingam E (2014). Emerging cystic fibrosis pathogens and the microbiome. Paediatr. Respir. Rev..

[4] Caraher E, Collins J, Herbert G, Murphy PG, Gallagher CG, Crowe MJ, Callaghan M, McClean S (2008). Evaluation of *in vitro* virulence characteristics of the genus *Pandoraea* in lung epithelial cells. J. Med. Microbiol..

[5] Costello A, Herbert G, Fabunmi L, Schaffer K, Kavanagh KA, Caraher EM, Callaghan M, McClean S (2011). Virulence of an Emerging Respiratory Pathogen, Genus *Pandoraea*, in vivo and Its Interactions with Lung Epithelial Cells. J. Med. Microbiol..

[6] Schneider I, Queenan AM, Bauernfeind A (2006). Novel carbapenem-hydrolyzing oxacillinase OXA-62 from *Pandoraea pnomenusa*. Antimicrob. Agents Chemother..

[7] Di Lorenzo F, De Castro C, Lanzetta R, Parrilli M, Silipo A, Molinaro A (2015). Carbohydrates in Drug Design and Discovery.

[8] Courtney JM, Ennis M, Elborn JS (2004). Cytokines and inflammatory mediators in cystic fibrosis. J. Cyst. Fibros..

[9] Molinaro A, Holst O, Di Lorenzo F, Callaghan M, Nurisso A, D’Errico G, Zamyatina A, Peri F, Berisio R, Jerala R, Jimenez-Barbero J, Silipo A, Martin-Santamaria S (2014). Chemistry of the lipid A: At the heart of innate immunity. Chem. Eur. J..

[10] Di Lorenzo F, Silipo A, Bianconi I, Lore’ NI, Scamporrino A, Sturiale L, Garozzo D, Lanzetta R, Parrilli M, Bragonzi A, Molinaro A (2015). Persistent cystic fibrosis isolate *Pseudomonas aeruginosa* strain RP73 exhibits an under-acylated LPS structure responsible of its low inflammatory activity. Mol. Immunol..

[11] Hamad MA, Di Lorenzo F, Molinaro A, Valvano MA (2012). Aminoarabinose is essential for lipopolysaccharide export and intrinsic antimicrobial peptide resistance in *Burkholderia cenocepacia*. Mol. Microbiol..

[12] Di Lorenzo F, Kubik Ł, Oblak A, Lorè NI, Cigana C, Lanzetta R, Parrilli M, Hamad MA, De Soyza A, Silipo A, Jerala R, Bragonzi A, Valvano MA, Martín-Santamaría S, Molinaro A (2015). Activation of Human Toll-like Receptor 4 (TLR4)·Myeloid Differentiation Factor 2 (MD-2) by Hypoacylated Lipopolysaccharide from a Clinical Isolate of Burkholderia cenocepacia. J. Biol. Chem..

[13] Maldonado RF, Sá-Correia I, Valvano MA (2016). Lipopolysaccharide modification in Gram-negative bacteria during chronic infection. FEMS Microbiol. Rev..

[14] Westphal O, Jann K (1965). Bacterial Lipopolysaccharides Extraction with Phenol-Water and Further Applications of the Procedure. Methods Carbohydr. Chem..

[15] Kittelberger R, Hilbink F (1993). Sensitive silver-staining detection of bacterial lipopolysaccharides in polyacrylamide gels. J. Biochem. Biophys. Methods.

[16] Rietschel ET (1976). Absolute configuration of 3-hydroxy fatty acids present in lipopolysaccharides from various bacterial groups. Eur. J. Biochem..

[17] Bligh EG, Dyer WJ (1959). A rapid method of total lipid extraction and purification. Can. J. Biochem. Physiol..

[18] Struwe WB, Agravat S, Aoki-Kinoshita KF, Campbell MP, Costello CE, Dell A, Feizi T, Haslam SM, Karlsson NG, Khoo KH, Kolarich D, Liu Y, McBride R, Novotny MV, Packer NH, Paulson JC, Rapp E, Ranzinger R, Rudd PM, Smith DF, Tiemeyer M, Wells L, York WS, Zaia J (2016). Kettner, C. The minimum information required for a glycomics experiment (MIRAGE) project: sample preparation guidelines for reliable reporting of glycomics datasets. Glycobiology.

[19] Di Lorenzo, F., Sturiale, L., Palmigiano, A., Fazio, L.L., Paciello, Ida, Coutinho, C.P., Sá-Correia, I., Bernardini, M.L., Lanzetta, R., Garozzo, D., Silipo, A., Molinaro, A.: Chemistry and biology of the potent endotoxin from a *Burkholderia dolosa* clinical isolate from a cystic fibrosis patient. ChemBioChem **14**, 1105–1115 (2013) 10.1002/cbic.201300062 23733445

[20] De Castro C, Parrilli M, Holst O, Molinaro A (2010). Microbe-associated molecular patterns in innate immunity: Extraction and chemical analysis of gram-negative bacterial lipopolysaccharides. Methods Enzymol..

[21] Silipo A, Lanzetta R, Amoresano A, Parrilli M, Molinaro A (2002). Ammonium hydroxide hydrolysis: a valuable support in the MALDI-TOF mass spectrometry analysis of Lipid A fatty acid distribution. J. Lipid Res..

[22] Phillips NJ, Schilling B, McLendon MK, Apicella MA, Gibson BW (2004). Novel Modification of Lipid A of *Francisella tularensis*. Infect. Immun..

[23] Barrau, C., Di Lorenzo, F., Menes, R.J., Lanzetta, R., Molinaro, A., Silipo, A.: The Structure of the Lipid A from the Halophilic Bacterium *Spiribacter salinus* M19-40^T^. Mar. Drugs. **16**(4), 124 (2018) 10.3390/md16040124 PMC5923411 29641496

[24] Pallach M, Di Lorenzo F, Duda KA, Le Pennec G, Molinaro A, Silipo A (2019). The Lipid A Structure From the Marine Sponge Symbiont *Endozoicomonas* Sp. HEX 311. Chembiochem.

[25] Di Lorenzo F, Palmigiano A, Bitar-Nehme A, Sturiale S, Duda L, Gully KA, Lanzetta D, Giraud R, Garozzo E, Bernardini D, Molinaro ML, Silipo A (2017). The Lipid A from *Rhodopseudomonas palustris* strain BisA53 LPS possesses a unique structure and low immunostimulant properties. Chemistry.

[26] Larrouy-Maumus G, Clements A, Filloux A, McCarthy RR, Mostowy S (2016). Direct detection of lipid A on intact Gram-negative bacteria: by MALDI-TOF mass spectrometry. J. Microbiol. Methods.

[27] Domon B, Costello CE (1988). A systematic nomenclature for carbohydrate fragmentations in FAB-MS/MS spectra of glycoconjugates. Glycoconjugate J..

[28] Kokcha S, Bittar F, Reynaud-Gaubert M, Mely L, Gomez C, Gaubert JY (2013). *Pandoraea pulmonicola* chronic colonization in a cystic fibrosis patient, France. New Microbes New Infect..

[29] Leung LM, Cooper VS, Rasko DA, Guo Q, Pacey MP, McElheny CL, Mettus RT, Yoon SH, Goodlett DR, Ernst RK, Doi YJ (2017). Structural modification of LPS in colistin-resistant, KPC-producing *Klebsiella pneumoniae*. Antimicrob. Chemother..

[30] Marr, N., Tirsoaga, A., Blanot, D., Fernandez, R., Caroff, M.: Glucosamine Found as a Substituent of Both Phosphate Groups in *Bordetella* Lipid A Backbones: Role of a BvgAS-Activated ArnT Ortholog. J. Bacteriol. 4281–4290 (2008) 10.1128/JB.01875-07 PMC2446747 18424515

[31] Preston A, Maxim E, Toland E, Pishko EJ, Harvill ET, Caroff M, Maskell DJ (2003). *Bordetella bronchiseptica* PagP is a Bvg-regulated lipid A palmitoyl transferase that is required for persistent colonization of the mouse respiratory tract. Mol. Microbiol..

[32] Pilione MR, Pishko EJ, Prestonm A, Maskellm DJ, Harvill ET (2004). pagP is required for resistance to antibody-mediated complement lysis during *Bordetella bronchiseptica* respiratory infection. Infect. Immun..

[33] Di Lorenzo, F., Silipo, A., Costello, A., Sturiale, L., Garozzo, D., Callaghan, M., Lanzetta, R., Parrilli, M., McClean, S., Molinaro, A.: Structural Study of the Lipopolysaccharide O-Antigen Produced by the Emerging Cystic Fibrosis Pathogen *Pandoraea pulmonicola*. Eur. J. Org. Chem. 2243–2249 (2012)

