# Supplementary material for: A chronic strain of the cystic fibrosis pathogen Pandoraea pulmonicola expresses a heterogenous hypo-acylated lipid A
Source: Glycoconj J. 2020 Oct 13;38(2):135–44. doi: 10.1007/s10719-020-09954-8 (PMC8052242; doi:10.1007/s10719-020-09954-8)

Supplementary Material

A chronic strain of the cystic fibrosis pathogen *Pandoraea pulmonicola* expresses a heterogenous hypo-acylated lipid A

Molly D. Pither,[1] Siobhán McClean,[2,3] Alba Silipo,[1] Antonio Molinaro,[1] Flaviana Di Lorenzo[1*]

[1]Department of Chemical Sciences, University of Naples Federico II, via Cinthia 4, 80126 Naples, Italy.

[2]Centre of Microbial Host Interactions, Institute of Technology Tallaght, Dublin, 24,

[3]School of Biomolecular and Biomedical Sciences, University College Dublin, Belfield, Dublin, 4, Ireland.

*****flaviana.dilorenzo@unina.it orcid.org/0000-0003-4821-0114

Siobhán McClean orcid.org/0000-0001-6389-2542
Antonio Molinaro orcid.org/0000-0002-3456-7369
Alba Silipo orcid.org/0000-0002-5394-6532

Fig S-1 Negative ion MALDI-TOF (reflectron mode) mass spectrum recorded directly on bacterial pellet of *P. pulmonicola* RL8228. "**P**" indicates the phosphate group; "**HexN**" indicates differences of 161 amu (i.e. a hexosamine unit).

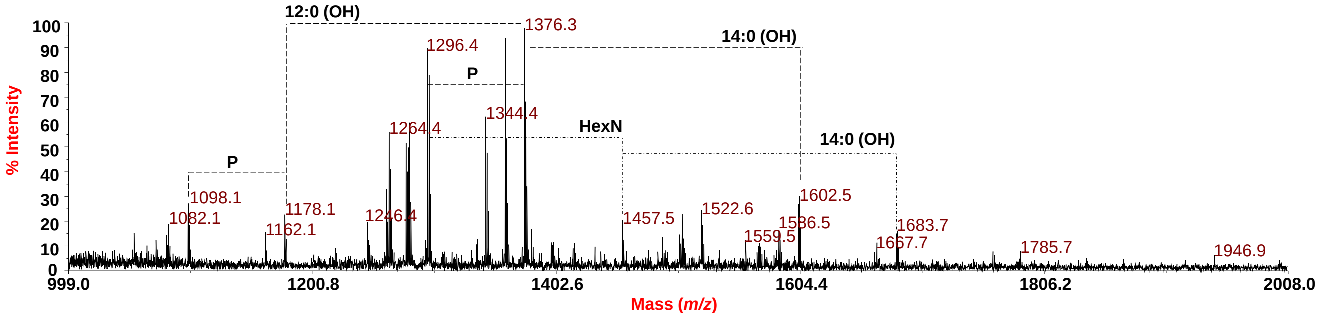

Fig S-2 Zoom of the negative-ion MALDI MS^2 spectrum of precursor ion at *m/z* 1457.5 of the lipid A isolated from *P. pulmonicola* RL8228. The spectrum shows the occurrence of an ion (*m/z* 240.12) matching with HexN-PO_3 loss, as shown in the sketched structure

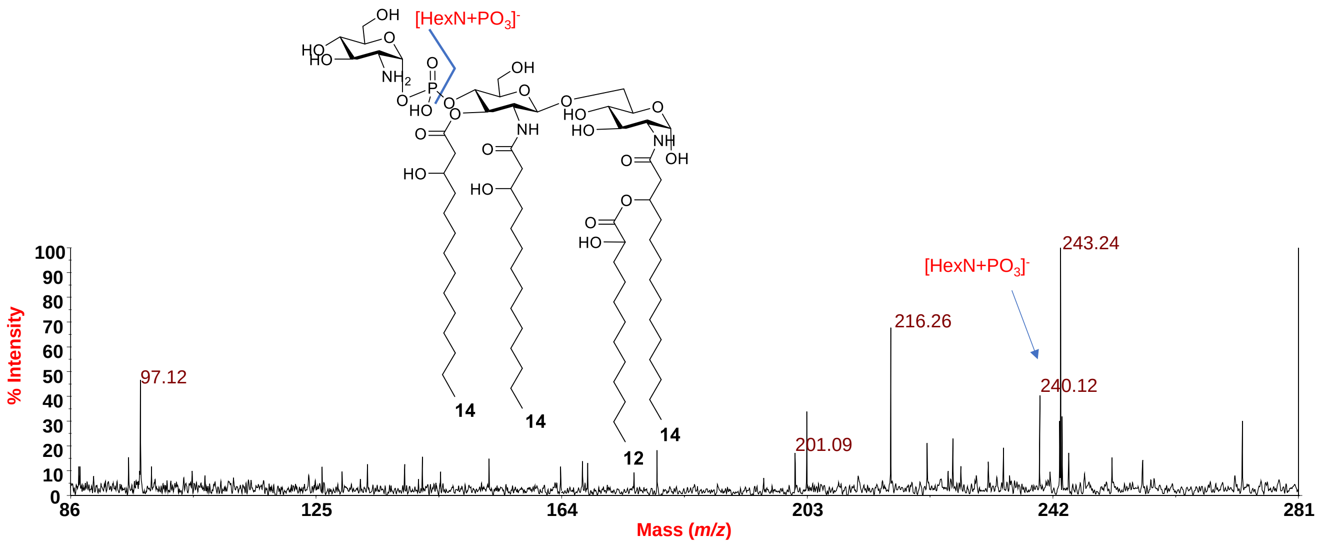

Fig S-3 a) The GC-MS chromatogram profile of the alditol acetate derivative of the HexN isolated from the *P. pulmonicola* RL8228 lipid A by de-phosphorylation and proper extraction. **b-d)** The GC-MS chromatogram profiles of the glucosaminitol, (**b**), galactosaminitol (**c**) and mannosaminitol (**d**) acetates opportunely prepared from authentical standards and used as a reference. The comparison of the retention times clearly proved that the unkown HexN decorating the lipid A of *P. pulmonicola* RL8228 was a GlcN. The black arrow indicates the peak relative to the uknown hexosaminitol acetate, whereas the blue cross indicates a contaminant.

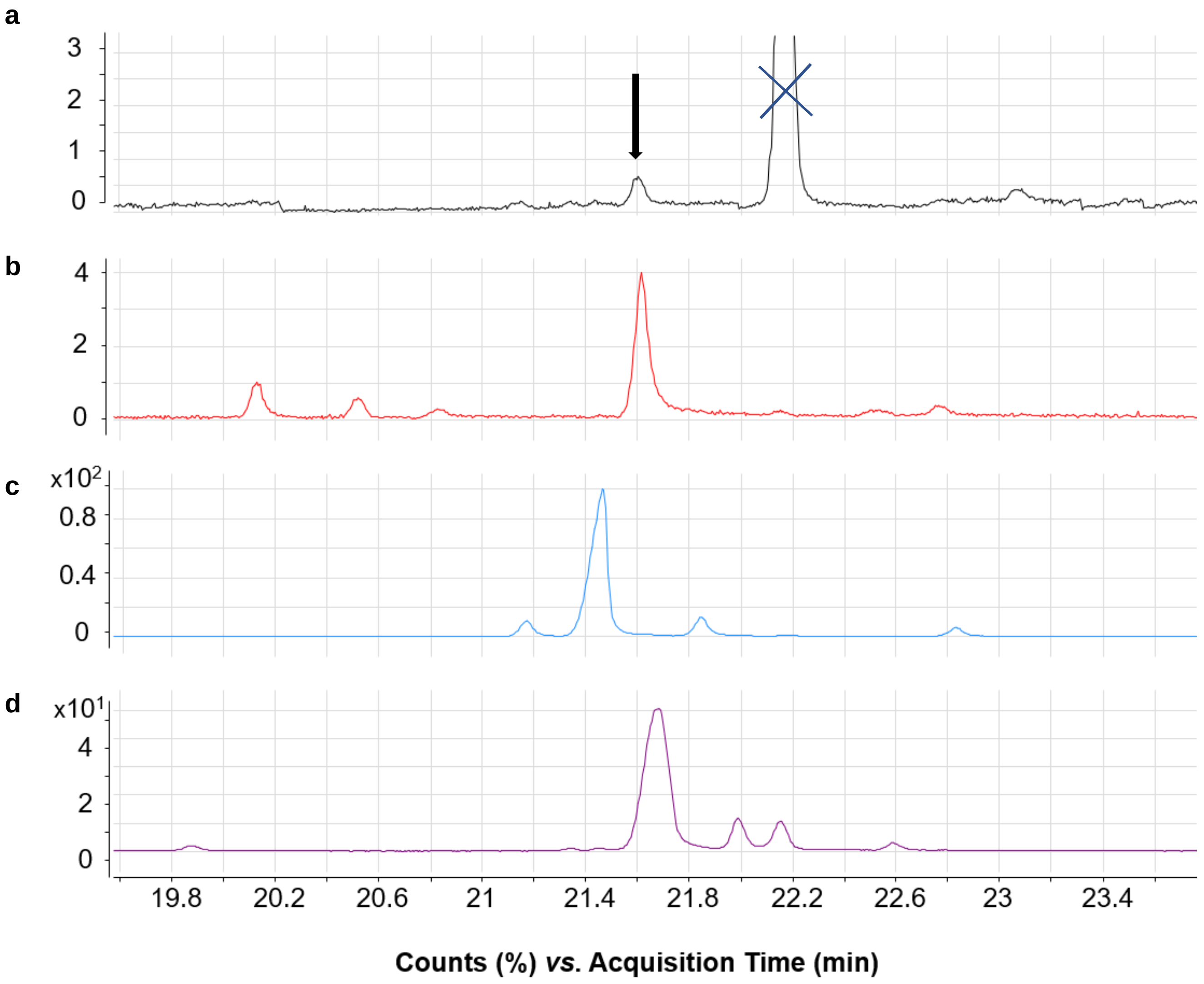

Supplement: Supplementary file 1 — (PDF 433 kb) [file 10719_2020_9954_MOESM1_ESM.pdf]
